# Persistent apogeotropic positional nystagmus of the lateral semicircular canal: clinical features and association with vestibular atelectasis on delayed 3D-FLAIR MRI

**DOI:** 10.3389/fneur.2026.1890082

**Published:** 2026-08-07

**Authors:** Louis Calvet, Edwin Regrain, Laurent Seidermann, Marc Labrousse, Xavier Dubernard

**Affiliations:** 1 Department of Otorhinolaryngology, CHU Reims, University of Reims Champagne-Ardenne, Reims, France; 2 Department of Dizziness, Balance and Gait Assessment and Rehabilitation (CERVEM), Courlancy Group – Les Bleuets Polyclinic, Reims, France

**Keywords:** apogeotropic positional nystagmus, delayed 3D-FLAIR MRI, lateral semicircular canal dysfunction, positional vertigo, vestibular atelectasis

## Abstract

**Objective:**

Persistent apogeotropic positional nystagmus of the lateral semicircular canal, often classified as atypical lateral semicircular canal (SCC) BPPV (alBPPV), may occur without typical positional nystagmus or response to repositioning maneuvers, in the absence of neurological disease. We aimed to describe its clinical and radiological features and investigate the occurrence and clinical correlates of utricular/saccular atelectasis on delayed 3D-FLAIR MRI.

**Methods:**

Retrospective single-center study in a tertiary referral center. Patients underwent clinical, vestibular, auditory and electrophysiological assessment, including videonystagmography, video head impulse testing, and delayed 3D-FLAIR MRI.

**Results:**

Thirty-two patients (mean age 68 years, vertigo duration 6.8 years) were included. Apogeotropic nystagmus persisted in 75%, with 50% meeting cupulolithiasis criteria. Geotropic conversion occurred in 15, and 10% showed no nystagmus. Vestibular investigations did not reveal a consistent or homogeneous vestibular deficit pattern. No consistent auditory abnormality was identified. Vibration-induced and head-shaking nystagmus were present in 30 and 50%, respectively, with kinetic hyporeflexia in 30%. Among patients with interpretable delayed 3D-FLAIR MRI, vestibular atelectasis was identified in approximately 60%. No significant clinical, audiological, or vestibular differences were found between patients with and without atelectasis.

**Conclusion:**

Persistent apogeotropic positional nystagmus involving the lateral SCC in the absence of neurological signs may represent a heterogeneous vestibular disorder. Vestibular atelectasis was commonly observed among patients with interpretable delayed 3D-FLAIR MRI, although its clinical and pathophysiological significance remains uncertain.

## Introduction

Benign paroxysmal positional vertigo (BPPV) affects 0.6% of the population annually and accounts for 8% of moderate-to-severe vertigo cases (1).

While BPPV’s pathophysiology remains debated (2), the condition is widely attributed to canalithiasis free-floating otoconia within the semicircular canal endolymph, leading to transient rotatory vertigo lasting a few minutes and positional nystagmus specific to the affected canal. Video nystagmography (VNG) assessment helps identify the affected semicircular canal (SCC) and side, in the absence of clinical neurological signs. Diagnostic criteria have been established by the Bárány Society (2).

Canalithiasis most commonly affects the posterior SCC, followed by lateral SCC (3), and only rarely the anterior SCC (4). Since the first descriptions of horizontal canal BPPV and its positional nystagmus variants by Pagnini et al. (3), atypical forms of lateral SCC positional vertigo have been increasingly recognized.

Typical lateral SCC involvement is characterized by purely horizontal, fatigable nystagmus with short latency (a few seconds) after positioning the patient along the canal axis. During anteflexion, the direction of the nystagmus helps identify the affected side. In the supine position, the nystagmus reverses and becomes geotropic in the lateral decubitus position. These findings are classically observed during the supine head-roll test used for the diagnosis of lateral SCC BPPV.

However, lateral SCC involvement may also present atypically (3), with prolonged, recurrent, or persistent manifestations. Nystagmus may be horizontal and atypical, without latency, with gradual fatigue exceeding 1 min, or may persist indefinitely. It can be geotropic or apogeotropic in the lateral decubitus position and standard therapeutic maneuvers may be ineffective. These features define a subgroup of persistent apogeotropic positional vestibular disorders, commonly classified as atypical BPPV of the lateral SCC (alBPPV), although its pathophysiology remains unclear. Even in the absence of signs suggesting a central cause, the clinico-radiological phenotype remains insufficiently characterized, leading to diagnostic and therapeutic challenges for clinicians (5). This study aimed to examine the epidemiological, clinical, electrophysiological, and radiological characteristics of patients with alBPPV in the absence of identified clinical and/or radiological neurological signs on MRI. We hypothesized that persistent apogeotropic positional nystagmus of the lateral SCC, when unresponsive to repositioning maneuvers and not explained by identified neurological disease, may involve vestibular mechanisms distinct from typical canalithiasis.

## Materials and methods

We conducted a descriptive, retrospective, single-center study of patients with atypical lateral semicircular canal BPPV (alBPPV) enrolled between September 2022 and November 2023 at the ENT department of a French tertiary care center.

As this was a retrospective study, eligible patients were identified from routine clinical records. Inclusion criteria were age ≥18 years, symptomatic alBPPV, defined by recurrent positional vertigo lasting more than 1 min and bilateral horizontal apogeotropic positional nystagmus observed in both right and left lateral decubitus positions on VNG, absence of clinical neurological signs, absence of radiological signs suggestive of a central neurological cause on brain MRI and failure of at least two modified Gufoni repositioning maneuvers.

Exclusion criteria were age <18 years, pregnancy, legal protection status, including curatorship or guardianship, absence of health insurance, clinical or MRI findings suggestive of a central cause, and incomplete clinical or vestibular data preventing confirmation of the diagnostic criteria.

Our primary objective was to describe the epidemiological, clinical, electrophysiological, and radiological characteristics of this population during electrophysiological testing.

Our secondary objective was to investigate whether identifying unilateral and/or bilateral utricular and/or saccular atelectasis on delayed 3D-FLAIR inner ear magnetic resonance imaging (MRI) would enable better phenotyping in this population.

Persistent apogeotropic positional vestibular disorder was defined based on clinical and VNG criteria. Patients had to experience recurrent positional vertigo lasting longer than 1 min, without neurological signs.

Neurological examination excluded ocular motor disorders, including ophthalmoplegia, saccadic and pursuit abnormalities, cranial nerve deficits, cerebellar dysfunction, lower-limb sensory deficits, and current clinical features suggestive of migraine or vestibular migraine.

### Vestibular, electrophysiological, and radiological assessment

All included patients underwent a standardized vestibular and electrophysiological assessment as part of routine clinical care. VNG was performed first and included assessment of spontaneous or pseudo-spontaneous nystagmus, positional testing in anteflexion, supine, right lateral decubitus, and left lateral decubitus positions, vibration-induced nystagmus, head-shaking test, kinetic testing, sweep and step tests, caloric testing, screening for central vestibular signs and bilateral vestibular areflexia. vHIT was then performed during the same session to assess high-frequency vestibulo-ocular reflex function in the six semicircular canals. Examinations were performed under standardized conditions by the same team using the Ulmer Synapsys Inventis® VNG system and the Ulmer III Synapsys Inventis® vHIT system according to the manufacturers’ protocols. Otolith function was assessed separately using c-VEMP when interpretable, also using the Echodia® system. However, c-VEMP responses were frequently absent or bilateral and were therefore not used for reliable side-specific interpretation. o-VEMP was not available during the study period.

For inclusion, VNG had to confirm horizontal apogeotropic positional nystagmus in both right and left lateral decubitus positions, persisting or becoming fatigable only after more than 1 min. The modified Gufoni maneuver had to fail at least twice. Cupulolithiasis was defined as bilateral horizontal apogeotropic nystagmus with an inter-side slow-phase velocity asymmetry of at least 25% and persistence for at least 1 min in one lateral decubitus position. This 25% asymmetry threshold was used as a predefined operational criterion in this study to identify a robust and reproducible side-to-side difference in nystagmus intensity. Although persistent bilateral apogeotropic horizontal nystagmus and side-to-side intensity differences are commonly used to support the diagnosis of horizontal canal cupulolithiasis, no universally validated percentage threshold is available.

Abnormal vHIT gain was defined as <0.71 for the lateral SCC, <0.81 for the superior SCC, and <0.71 for the posterior SCC. Positive vibration-induced nystagmus was defined as a slow-phase velocity >5°/s. Directional preponderance was considered positive when >3°/s during sinusoidal testing and reproducible across kinetic conditions. Kinetic hyporeflectivity was defined as VOR gain ≤0.4 at 0.1 Hz and 0.64 Hz sinusoidal testing with absence of an all-pass profile on sweep testing. Caloric hypovalence was categorized as 20–50% or >50%.

Auditory and cochlear electrophysiological assessment included pure-tone and speech audiometry, electrocochleography, and DPOAE phase-shift analysis. Electrocochleography and DPOAE phase-shift analysis were performed using the Echodia® system. Electrocochleography was used to assess the SP/AP ratio as an indirect marker of inner ear pressure abnormalities, with an SP/AP ratio >40% considered abnormal. DPOAE phase-shift analysis was used to explore cochlear micromechanical changes potentially associated with endolymphatic pressure variations.

Brain MRI and contrast-enhanced cerebellopontine angle MRI were performed to exclude a central neurological cause or retrocochlear lesion. In our clinical practice, delayed 3D-FLAIR inner ear MRI is proposed in complex vestibular presentations, particularly when symptoms are atypical, recurrent, persistent, or resistant to standard treatment, including atypical positional vertigo. When technically feasible and accepted by the patient, this imaging is used to exclude central or retrocochlear causes and to assess inner ear abnormalities, including endolymphatic hydrops and vestibular atelectasis.

All brain and inner ear MRI scans were performed using the same 1.5-Tesla scanner and were reviewed by two expert radiologists, two ENT specialists, and a rehabilitation physician. Vestibular atelectasis was radiologically defined as collapse of the utricular and/or saccular endolymphatic space with inward deformation of the membranous labyrinth. Patients with interpretable delayed 3D-FLAIR inner ear MRI were categorized into two groups according to the absence or presence of vestibular atelectasis: Group 1 included patients without vestibular atelectasis (*n* = 7), and Group 2 included patients with vestibular atelectasis (*n* = 12).

A database (Excel file) was used to compile epidemiological, audiometric, electrophysiological, vestibular, and radiological data. Collected data included demographic characteristics, medical history, vertigo history and symptom duration, DHI score when available, otological symptoms, pure-tone and speech audiometry, VNG and vHIT parameters, caloric and kinetic test results, electrocochleography, DPOAE phase-shift analysis, and radiological findings on brain and delayed 3D-FLAIR inner ear MRI. Quality of life related to dizziness was assessed using the Dizziness Handicap Inventory (DHI), when available, at the time of the vestibular assessment, after failure of repositioning maneuvers. The DHI was not administered before the initial maneuvers and was not used as a longitudinal outcome measure.

This retrospective observational study was reported in accordance with the Strengthening the Reporting of Observational Studies in Epidemiology (STROBE) guidelines. It was conducted in accordance with French regulations and complied with the CNIL MR-004 reference methodology for research involving the reuse of health data collected during routine clinical care. Patients were informed that their anonymized data could be used for research purposes and could object to or withdraw from such use. All procedures were conducted in accordance with the principles of the Declaration of Helsinki and Good Clinical Practice guidelines.

### Statistical analysis

Descriptive analyses were performed for the overall cohort. No *a priori* sample size calculation was performed because this was a retrospective, descriptive, exploratory study. The sample size was determined by the number of eligible patients identified from routine clinical records during the study period. Continuous variables were expressed as means and medians, and categorical variables as numbers and percentages. Comparisons between patients with and without vestibular atelectasis on delayed 3D-FLAIR inner ear MRI were performed as exploratory analyses. Categorical variables were compared using Fisher’s exact test, and continuous variables were compared using the non-parametric Mann–Whitney U test. Statistical analyses were performed using StatPlus software. A *p*-value <0.05 was considered statistically significant.

## Results

### General population study

#### Epidemiology

**Figure 1:**
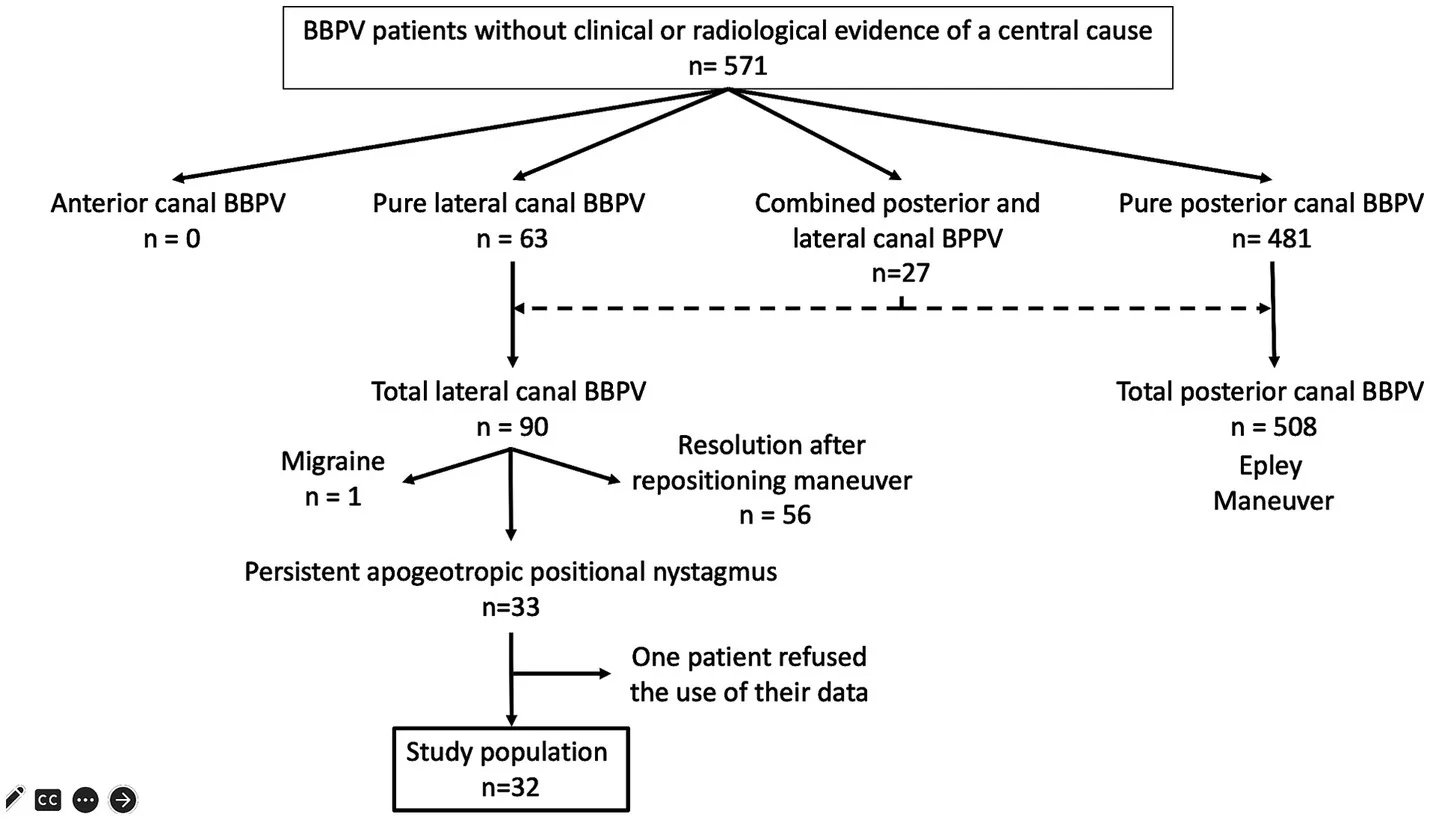
Flow chart of patient selection. BPPV, benign paroxysmal positional vertigo.

The patient selection process is summarized in Figure 1. Table 1 presents the epidemiological characteristics of the patients (*n* = 32) included consecutively over a 14-month period. The cohort was predominantly female (71.9% vs. 28.1%) and the mean age at diagnosis was 68.5 [37–84] years. A history of cardiovascular disease was reported in 46.9% of the patients. Clinical examination did not reveal neurological disorders and otoscopy was consistently normal.

**Table 1:** Epidemiological and clinical characteristics of the general population.

Epidemiological and clinical data	
Female sex	23 (71.9%)
Male sex	9 (28.1%)
Average age (years)	68.5 [37–84]
Medical history	
- Cardiovascular	15 (46.9%)
- Cancer	6 (18.8%)
- Thyroid disease	6 (18.8%)
- Anxiety disorders	5 (15.6%)
- History of migraine	2 (6.3%)
History of vertigo	
- BPPV	32 (100%)
- Prolonged vertigo	19 (59.4%)
- Repositioning maneuvers	28 (87.5%)
- Average number of maneuvers	4.7 [0–20]
- History of vestibular rehabilitation	5 (15.6%)
- Average interval between 1st vertigo and VNG (years)	6.8 [0–25]
Current vertigo	
Episode duration <3 min	26 (81.3%)
Episode duration >20 min	6 (18.7%)
DHI score	31.5 [7–82]
- DHI < 40	22 (68.8%)
- DHI > 40	10 (31.2%)
Mean interval between last attack and inclusion (days)	27 [1–230]
Mean interval between inclusion and VNG (days)	15 [1–100]
Mean interval between last attack and VNG (days)	42 [2–231]
Mean interval first attack and VNG (days)	118 [5–360]
Symptomatic on day of VNG	22 (68.6%)
Tinnitus	
- Bilateral	8 (25%)
- Unilateral	8 (25%)
- None	16 (50%)
Perceived hearing loss	
- Bilateral	2 (6.3%)
- Unilateral	3 (9.4%)
- None	27 (84.3%)
Aural fullness	
- Bilateral	0 (0%)
- Unilateral	7 (21.9%)
- None	25 (78.1%)
Otophonia	0 (0%)
Neurovegetative symptoms	11 (34.4%)
Vestibular deviation	0 (0%)
Menière’s triad	2 (6.3%)
Cerebellar syndrome	0 (0%)
Cranial nerve disorder	0 (0%)
Normal otoscopy	32 (100%)

BPPV, benign paroxysmal positional vertigo; DHI, Dizziness Handicap Inventory.

Menière’s triad: Combination of vertigo lasting more than 20 min, unilateral tinnitus and unilateral hearing loss.

#### Description of vertigo

All included patients experienced at least one episode of BPPV. On average, 87.5% of the patients were treated with therapeutic maneuvers (average: 4.7) and vestibular rehabilitation was prescribed in 15.6% of cases. None of these treatments resulted in sustained symptom resolution.

The persistence of symptoms, the mean diagnostic delay of 6.8 years (interval between first vertigo episode and inclusion), prolonged vertigo in 59.4% of cases (average duration: 1.6 h) and the DHI results obtained at the time of vestibular assessment suggested a substantial perceived functional burden in these patients. Approximately 31% of patients had a Dizziness Handicap Inventory score >40. Monthly vertigo episodes averaged 14 (3.5 per week). On average, the most recent episode occurred within 27 days preceding study inclusion. An inverse relationship existed between attack duration and frequency (0.8 vs. 16.8 episodes; *p* = 0.04).

Hearing loss, aural fullness, and tinnitus were observed in 15.7, 21.9, and 50% of cases, respectively. Only 6.3% of the patients exhibited a clinical symptom triad suggestive of Menière disease.

#### Vestibular and auditory electrophysiological investigations

The mean interval from enrollment to examination was 15 days (median, 9 days), corresponding to 42 days from the last episode to examination (Table 2).

**Table 2:** Electrophysiological and audiometric characteristics of the general population.

Electrophysiological and audiometric data	
vHIT	32 (100%)
- Lateral right SCC average VOR gain	0.95
- Lateral left SCC average VOR gain	0.98
- Anterior right SCC average VOR gain	0.98
- Anterior left SCC average Gain VOR	0.95
- Posterior right SCC average Gain VOR	0.83
- Posterior left SCC average VOR gain	0.83
- Abnormal right lateral SCC	2 (6.3%)
- Abnormal left lateral SCC	0 (0%)
- Anterior right SCC abnormal	0 (0%)
- Anterior left SCC abnormal	0 (0%)
- Abnormal right posterior SCC	5 (15.6%)
- Abnormal left posterior SCC	5 (15.6%)
Spontaneous nystagmus	7 (21.9%)
- Grade 1	2 (28.6%)
- Grade 2	3 (42.8%)
- Grade 3	2 (28.6%)
- Mean velocity (°/s)	0.54 [0.4–1]
- Maximum velocity (°/s)	1.49 [0.6–3]
- Consistency with positive HST	7 (100%)
Nystagmus HST	17 (53.1%)
- Average velocity (°/s)	3.68 (2.4)
- Maximum velocity (°/s)	7.31 (6.3)
Positive VIN (>5°/s)	9 (28.1%)
HST and/or VIN positive nystagmus	18 (56.3%)
- HST and VIN consistent	4 (22.2%)
- HST and VIN inconsistent	4 (22.2%)
- HST positive alone	9 (50%)
- VIN positive alone	1 (5.6%)
Nystagmus Anteflexion	14 (43.8%)
- Average velocity (°/s)	1.75 [0.2–13.8]
- Maximum velocity (°/s)	3.08 [0.5–17.1]
Supine nystagmus	13 (40.6%)
- Average velocity (°/s)	2.7 [0.3–17.5]
- Maximum velocity (°/s)	5.04 [0.7–22]
Nystagmus Anteflexion and/or positive supine position	19 (59.4%)
- Reversal	3 (15.8%)
- Without reversal	6 (31.6%)
- Positive anteflexion only	5 (26.3%)
- Positive dorsal decubitus only	5 (26.3%)
Nystagmus Right lateral decubitus	29 (90.6%)
- Average velocity (°/s)	8.78 [0.3–32.2]
- Maximum velocity (°/s)	11.94 [0.9–33.7]
- Vertigo	9 (31%)
- Fatigable	3 (10.3%)
- Decreasing over time	10 (34.5%)
Nystagmus Left lateral decubitus	29 (90.6%)
- Average velocity (°/s)	8.43 [0.4–32.5]
- Maximum velocity (°/s)	12.06 [0.9–60.9]
- Vertigo	9 (31%)
- Fatigable	3 (10.3%)
- Decreasing over time	10 (34.5%)
Apogeotropic nystagmus	24 (75%)
Geotropic nystagmus	5 (15.6%)
No nystagmus	3 (9.4%)
Cupulolithiasis	15 (46.9%)
- Right	6 (40%)
- Left	9 (60%)
Kinetics (sinusoid 0.1 Hz)	32 (100%)
- Gain	0.38 [0.09–0.64]
- Gain < 0.4	18 (56.3%)
- Gain < 0.2	2 (6.3%)
- Preponderance > 3°/s	4 (12.5%)
Kinetic (sinusoid 0.64 Hz)	32 (100%)
- Gain	0.42 [0.18–0.71]
Kinetics (sweep)	32 (100%)
- All-pass	15 (46.9%)
- Low-pass	12 (37.5%)
- No-pass profile	1 (3.1%)
- High-pass	3 (9.4%)
- Low-frequency attenuation	1 (3.1%)
Kinetics (slots)	32 (100%)
Kinetics (Step test)	23 (71.9%)
- Normal time constant	23 (100%)
Sine wave 0.1 Hz, 0.64 Hz and sweep < 0.4	9 (28.1%)
Caloric	32 (100%)
- Cumulative caloric reflectivity	53.57 [15.3–137.6]
- Absolute preponderance > 3	5 (15.6%)
- No hypovalence	17 (53.1%)
- Hypovalence > 20 and < 50	10 (31.3%)
- Hypovalence > 50	5 (15.6%)
VNG centrality criteria	0 (0%)
Bilateral vestibular areflexia criteria	0 (0%)
DPOAE phase-shift analysis	30 (93.8%)
- No dephasing	25 (83.3%)
- Unilateral dephasing	5 (16.7%)
ECoG with bilateral wave I response	32 (100%)
- Normal SP/AP	28 (87.5%)
- Unilateral SP/AP ratio >40%	4 (12.5%)
Pure-tone audiometry (dB)	32 (100%)
- Asymmetrical right/left PTA	3 (9.4%)
- Asymmetrical right/left low-frequency PTA	2 (6.2%)
- Normal hearing	16 (50%)
- Mild bilateral hearing loss	6 (18.8%)
- Moderate bilateral hearing loss	2 (6.3%)
- Unilateral mild hearing loss	5 (15.6%)
- Unilateral moderate hearing loss	2 (6.3%)
- Mild and moderate hearing loss	1 (3.1%)
Speech audiometry	32 (100%)
- Right speech intelligibility > 50% at 30 dB	22 (68.8%)
- Left speech intelligibility > 50% at 30 dB	27 (84.4%)
Vocal tonal dissociation	12 (37.5%)
- Bilateral	1 (8.3%)
- Unilateral	11 (91.7%)

VHIT, video head impulse test; VOR, vestibulo-ocular reflex; SCC, semicircular canal; HST, head shaking test; VIN, vibration-induced nystagmus; PTA, average hearing loss.

Regarding vestibular symptoms, 68.8% of patients remained symptomatic. Pseudospontaneous nystagmus was identified in 21.9% of cases. Although its characteristics were considered insignificant (mean velocity: 0.5°/s), 71% of the patients remained symptomatic and therefore, clinically relevant.

vHIT showed preserved VOR gains in most patients, with only isolated canal abnormalities and no consistent deficit pattern. Abnormal lateral SCC gain was observed in two patients, while posterior SCC gain abnormalities were observed in a limited number of cases. These abnormalities were not associated with a specific radiological profile.

VIN was positive in 28.1% of cases and HST in 53.1% (mean nystagmus velocity, 3.68°/s). In the case of positive VIN and/or HST results, only 22.2% of patients exhibited a consistent response to both tests (nystagmus beating in the same direction).

The kinetic tests revealed reproducible kinetic hyporeflectivity in 28.1% of cases (gain ≤0.4 in the VOR at 0.1 Hz and 0.64 Hz sinusoids and absence of all-pass profile in the sweep test). The directional preponderance was positive in 12% of cases (>3°/s in the 0.1 Hz sinusoids and reproducible in the 0.64 Hz sinusoids and in the niche), while the step test time constant remained normal in all patients.

In caloric testing, the mean total caloric reflectivity was 53°/s. Over 50% of patients exhibited no hypovalence.

In otolith testing, c-VEMP results were not usable in most patients.

In the lateral decubitus position tests, 75% of patients had apogeotropic nystagmus, 15.6% had geotropic nystagmus, and 9.4% had no nystagmus. Therefore, vestibular fluctuations were observed between the time of inclusion and VNG in 25% of the cases. Among patients presenting with the apogeotropic form (*n* = 24), only 15 had cupulolithiasis (46.9% of the general population).

In our study, 59.4% of patients (*n* = 19/32) had nystagmus in the anteflexion and/or supine position. Of these, 52.6% (*n* = 10/19) had horizontal nystagmus only in the anteflexion or supine position. Of the remaining 41.4% (*n* = 9/19), 30% had nystagmus that reversed between the two tested positions (*n* = 3/9). VNG showed no findings suggestive of central involvement or bilateral involvement.

Regarding hearing, 50% of patients had normal tonal audiometry. Speech audiometry was normal in 70% of cases on the right side and 85% on the left side. When hearing loss was present, it was almost never asymmetrical (9.4% of the patients). Electrocochleography (ECoG) and DPOAE phase-shift analysis were normal (87.5 and 83.3%, respectively).

#### MRI radiological characteristics of our population

**Figure 2:**
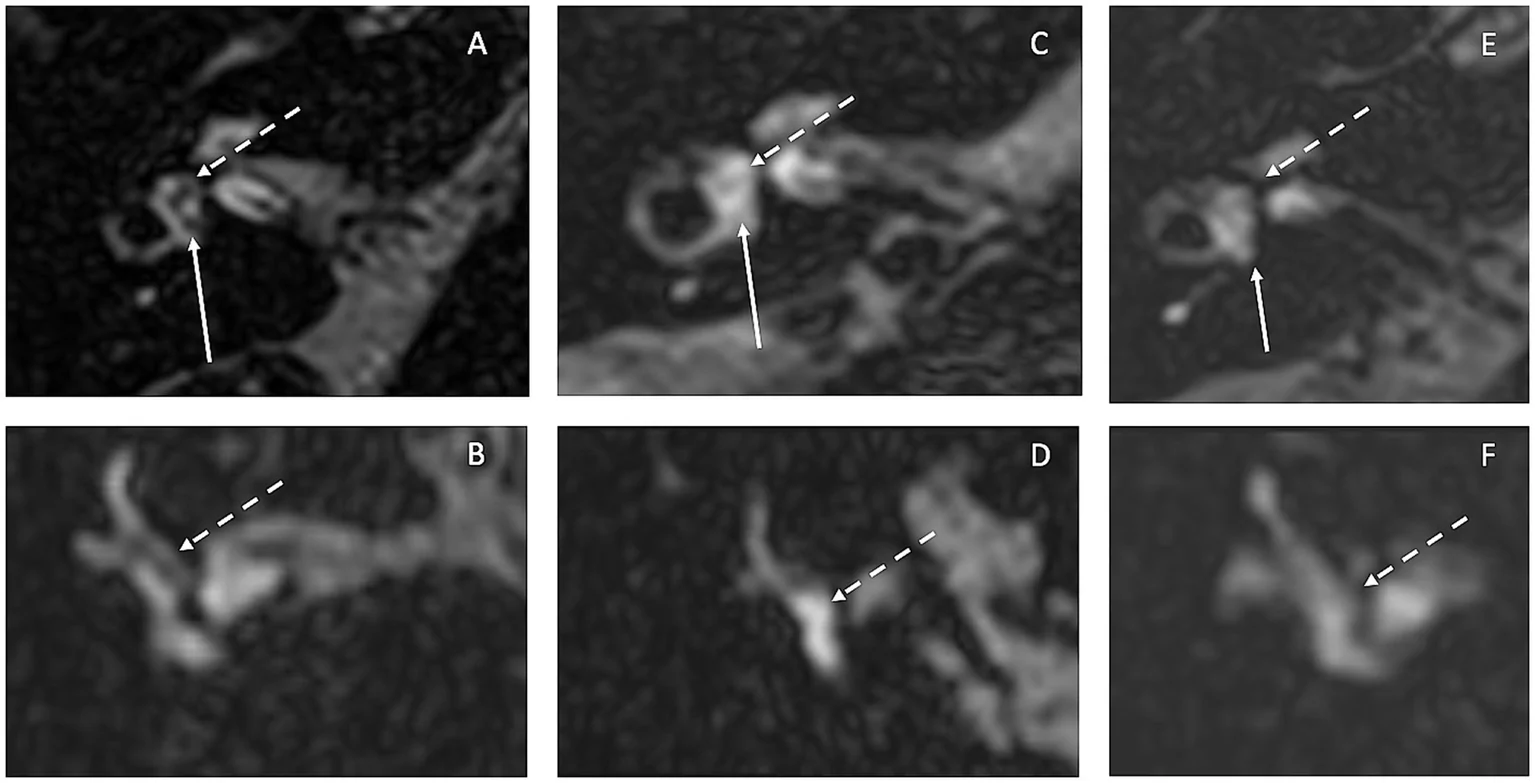
Vestibular atelectasis on delayed 3D-FLAIR inner ear MRI in persistent apogeotropic positional nystagmus. Delayed 3D-FLAIR inner ear MRI centered on the right ear, shown in axial **(A,C,E)** and oblique coronal **(B,D,F)** planes. **A,B** are from the same patient and show normal utricular and saccular morphology, with no evidence of hydrops or vestibular atelectasis. The solid arrow indicates the utricle, and the dashed arrow indicates the saccule, both structures are visible. **C–F** correspond to two different patients, each showing right vestibular atelectasis involving both the utricle and the saccule. Solid arrows indicate absence or collapse of the utricle, whereas dashed arrows indicate absence or collapse of the saccule.

All MRI images were acquired using a 1.5 Tesla device (Table 3). No brain MRI scans showed neurological abnormalities. Delayed 3D-FLAIR inner ear MRI was interpretable in 21 of 32 patients (65.6%). Nine patients could not complete the examination because of claustrophobia, and two examinations were uninterpretable because of motion artefacts. Among patients with evaluable imaging, seven examinations were normal (33.3%), and vestibular atelectasis was identified in 13 patients (62%) (Figure 2), including one patient with associated endolymphatic hydrops. Atelectasis was bilateral in approximately two-thirds of affected patients. Endolymphatic hydrops was identified in two patients, including one with associated atelectasis.

**Table 3:** Radiological characteristics of the general population.

Radiological data	
Normal brain MRI	32 (100%)
Interpretable delayed 3D-FLAIR inner ear MRI	21 (65.6%)
- Normal	7 (33.3%)
- Hydrops	1 (4.8%)
- Hydrops + atelectasis	1 (4.8%)
- Atelectasis	12 (57.1%)
- Bilateral utricle and saccule	3 (25%)
- Unilateral utricle and saccule	3 (25%)
- Utricle saccule and saccule	2 (16.7%)
- Bilateral saccule	2 (16.7%)
- Unilateral saccule	1 (8.3%)
- Bilateral utricle	1 (8.3%)
- Bilateral BHL disruption	1 (4.8%)
- Unilateral BHL disruption	3 (14.3%)
Interval between VNG and MRI (days)	27 (13)
Radiological analysis by clinical entity	
Cupulolithiasis	9 (28%)
- Atelectasis	5 (55.6%)
Bilateral	3 (60%)
Unilateral	2 (40%)
Coherent side	1 (20%)
- Normal	4 (44.4%)
No cupulolithiasis	12 (37.5%)
- Atelectasis	7 (58.3%)
- Bilateral	5 (71.4%)
- Unilateral	2 (28.6%)
- Hydrops	1 (8.3%)
- Atelectasis + hydrops	1 (8.3%)
- Normal	3 (25%)

BHL, blood-labyrinth barrier disruption.

In alBPPV cases not resembling cupulolithiasis, delayed 3D-FLAIR MRI findings did not differ significantly according to whether patients met the operational criteria for cupulolithiasis. Detailed findings are presented in Table 3.

### Epidemiological, clinical, vestibular, electrophysiological and audiometric comparisons of patients with (group 2) and without (group 1) atelectasis on delayed 3D-FLAIR inner ear

The presence of vestibular atelectasis was not significantly associated with any epidemiological, clinical, audiological, or vestibular variable assessed in this study (Table 4). Mean age was 64.4 years in patients with atelectasis and 69.4 years in those without atelectasis (*p* = 0.35). Cardiovascular comorbidities were present in 33 and 66% of patients, respectively (*p* = 0.16), while prolonged vertigo was reported in 41.7 and 71.4% (*p* = 0.34). A DHI score >40 was observed in 16.7% of patients with atelectasis and 42.9% of those without atelectasis (*p* = 0.30). Tinnitus was reported in 58.3 and 28.6% of patients, respectively (*p* = 0.35).

**Table 4:** Epidemiological, clinical, vestibular, electrophysiological and audiometric comparisons of patients with (group 2) and without (group 1) atelectasis on 3D FLAIR sequence delayed inner ear MRI.

Epidemiological, clinical, vestibular, electrophysiology and audiometric data	Group 1 (*n* = 7)	Group 2 (*n* = 12)	*p* value
Female sex	4 (57%)	9 (75%)	0.61
Male sex	3 (43%)	3 (25%)	0.61
Average age (years)	69.4 [50–79]	64.4 [37–82]	0.35
Medical history			
- Cardiovascular	5 (66%)	4 (33%)	0.16
- Cancer	3 (42.9%)	1 (8.3%)	0.11
- Thyroid disease	1 (14.3%)	2 (16.7%)	1
- Anxiety disorders	0 (0%)	1 (8.3%)	1
- Migraine	0 (0%)	1 (8.3%)	1
History of vertigo			
- BPPV	7 (100%)	12 (100%)	
- Long Vertigo	5 (71.4%)	5 (41.7%)	0.34
- Repositioning maneuvers	7 (100%)	10 (83.3%)	0.51
- Average number of maneuvers	6.5 [1–15]	5.3 [2–20]	0.67
- History of vestibular rehabilitation	1 (14.3%)	0 (0%)	0.37
Average interval between 1st vertigo and VNG (years)	9.75 (10)	6.6 (7.5)	0.29
Current vertigo			
Episode duration < 3 min	6 (85.7%)	9 (75%)	1
Episode duration > 20 min	1 (14.3%)	3 (25%)	1
DHI score	33.5 (34)	27 (31)	0.47
- DHI < 40	4 (57.1%)	10 (83.3%)	0.30
- DHI > 40	3 (42.9%)	2 (16.7%)	0.30
Mean interval between last dizzy spell and inclusion (days)	25.3 [1–75]	7.5 [1–30]	0.21
Mean interval between inclusion and VNG (days)	28.8 [3–100]	11.4 [6–30]	0.12
Mean interval between last attack of vertigo and VNG (days)	54 [8–109]	19 [8–51]	0.08
Tinnitus			
- Bilateral	1 (14.3%)	4 (33.3%)	0.35
- Unilateral	1 (14.3%)	3 (25%)	1
- None	5 (71.4%)	5 (41.7%)	0.35
Perceived hearing loss			
- Bilateral	1 (14.3%)	1 (8.3%)	1
- Unilateral	0 (0%)	1 (8.3%)	1
- None	6 (85.7%)	10 (83.3%)	1
Aural fullness			
- Bilateral	0 (0%)	0 (0%)	
- Unilateral	1 (14.3%)	2 (16.7%)	1
- None	6 (85.7%)	10 (83.3%)	1
Otophonia	0 (0%)	0 (0%)	
Neurovegetative symptoms	4 (57.1%)	6 (50%)	1
Vestibular deviation	0 (0%)	0 (0%)	
Coherent and positive triad	0 (0%)	0 (0%)	
Cerebellar syndrome	0 (0%)	0 (0%)	
Cranial nerve disorder	0 (0%)	0 (0%)	
Normal otoscopy	7 (100%)	12 (100%)	
vHIT	7 (100%)	12 (100%)	
- Unilateral lateral deficit	0 (0%)	2 (16.7%)	0.5
- Bilateral posterior deficit	1 (14.3%)	2 (16.7%)	0.84
Spontaneous nystagmus	0 (0%)	4 (25%)	0.25
HST nystagmus	4 (57.1%)	6 (50%)	1
Positive VIN (>5°/s)	2 (28.6%)	2 (16.7%)	0.60
HST and/or VIN positive nystagmus	4 (57.1%)	6 (50%)	1
- HST and VIN consistent	2 (50%)	1 (16.7%)	0.5
- HST and VIN inconsistent	0 (0%)	1 (16.7%)	1
- HST positive alone	2 (50%)	4 (66.7%)	1
- VIN positive alone	0 (0%)	0 (0%)	
Anteflexion nystagmus	4 (57.1%)	6 (50%)	1
Dorsal decubitus nystagmus	4 (57.1%)	6 (50%)	1
Positive anteflexion and/or supine nystagmus	5 (71.4%)	9 (75%)	1
- Reversal	1 (20%)	2 (22.2%)	1
- No reversal	2 (40%)	2 (22.2%)	0.58
- Positive anteflexion alone	1 (20%)	2 (22.2%)	1
- Positive dorsal decubitus alone	1 (20%)	3 (33.3%)	1
Nystagmus Right lateral decubitus	6 (85.7%)	11 (91.7%)	1
- Average velocity	11.01 [2.4–18.2]	14.04 [0.3–32.2]	0.66
- Maximum speed	14.78 [4.6–28.3]	17.55 [1.2–33.7]	0.3
- Vertigo	3 (50%)	5 (45.5%)	1
- Fatigable	0 (0%)	0 (0%)	
- Decreasing over time	2 (33.3%)	8 (72.7%)	0.16
Nystagmus Left lateral decubitus	6 (85.7%)	11 (91.7%)	1
- Average velocity	12.85 [0.6–26.8]	9.7 [0.4–32.5]	0.59
- Maximum velocity	15.47 [1.9–28.8]	14.85 [0.9–60.9]	0.66
- Vertigo	3 (50%)	4 (36.4%)	0.64
- Fatigable	0 (0%)	0 (0%)	
- Decreasing over time	2 (33.3%)	8 (72.7%)	0.16
Apogeotropic nystagmus	5 (71.4%)	9 (75%)	1
Geotropic nystagmus	1 (14.3%)	2 (16.7%)	1
No Nystagmus	1 (14.3%)	1 (8.3%)	1
Kinetics (sinusoid 0.1 Hz)	7 (100%)	12 (100%)	
- Gain	0.4 [0.1–0.62]	0.39 [0.09–0.64]	0.66
- Gain < 0.4	3 (42.9%)	5 (41.7%)	1
- Gain < 0.2	1 (14.3%)	1 (8.3%)	1
- Preponderance > 3°/s	1 (14.3%)	2 (16.7%)	1
Kinetic (sinusoid 0.64 Hz)	7 (100%)	12 (100%)	
- Gain	0.39 [0.21–0.65]	0.42 [0.18–0.71]	0.9
Kinetics (sweep)	7 (100%)	12 (100%)	
- All-pass	4 (57.1%)	5 (41.7%)	0.65
- Low-pass	2 (28.6%)	4 (33.3%)	0.52
- No-pass profile	1 (14.3%)	1 (8.3%)	1
- High-pass	0 (0%)	1 (8.3%)	1
- Low-pass	0 (0%)	1 (8.3%)	1
Kinetics (slots)	7 (100%)	12 (100%)	
Kinetics (Step test)	6 (85.7%)	8 (66.7%)	
- Normal time constant	6 (100%)	8 (100%)	
Sine wave 0.1 Hz, 0.64 Hz and sweep < 0.4	1 (14.3%)	3 (25%)	1
Caloric	7 (100%)	12 (100%)	
Cumulative reflectivity	59.2 [15.3–137.6]	49.2 [26.5–76.9]	0.6
Absolute preponderance > 3	1 (14.3%)	3 (25%)	1
No hypovalence	3 (42.9%)	5 (41.7%)	1
Hypovalence > 20 and < 50	1 (14.3%)	5 (41.7%)	1
Hypovalence > 50	1 (14.3%)	2 (16.7%)	1
VNG centrality criteria	0 (0%)	0 (0%)	
Bilateral vestibular areflexia criteria	0 (0%)	0 (0%)	
Dephasing of acoustic distortion products	7 (100%)	11 (91.7%)	
- No dephasing	7 (100%)	10 (90.9%)	1
- Unilateral dephasing	0 (0%)	1(9.1%)	1
ECoG with bilateral I-wave	7 (100%)	12 (100%)	
- Normal SP/AP	5 (71.4%)	11 (91.7%)	0.52
- Unilateral SP/AP ratio >40%	2 (28.6%)	1 (8.3%)	0.52
Pure-tone audiometry (dB)	7 (100%)	12 (100%)	
- Asymmetrical right/left PTA	1 (14.3%)	0 (0%)	0.37
- Asymmetrical right/left low-frequency PTA	0 (0%)	0 (0%)	
- Normal hearing	3 (42.9%)	7 (58.3%)	0.65
- Mild bilateral hearing loss	1 (14.4%)	3 (25%)	0.52
- Moderate bilateral hearing loss	0 (0%)	0 (0%)	
- Mild unilateral hearing loss	2 (28.7%)	2 (16.7%)	0.6
- Unilateral moderate hearing loss	0 (0%)	0 (0%)	
- Mild and moderate hearing loss	1 (14.3%)	0 (0%)	0.37
Speech audiometry	7 (100%)	12 (100%)	
- Right speech intelligibility >50% at 30 dB	6 (85.7%)	8 (66.7%)	0.6
- Left speech intelligibility >50% at 30 dB	6 (85.7%)	9 (75%)	1
Vocal tonal dissociation	1 (14.3%)	5 (41.6%)	0.33
- Bilateral	0 (0%)	0 (0%)	
- Unilateral	1 (100%)	5 (100%)	
Interval between VNG and MRI (days)	26 (5)	31 (17.5)	0.52

BPPV, benign paroxysmal positional vertigo; DHI, Dizziness Handicap Inventory; vHIT, video head impulse test; SCC, semicircular canal; HST, head shaking test; VIN, vibration-induced nystagmus; PTA, average hearing loss.

Meniere’s triad: Combination of vertigo lasting more than 20 min, unilateral tinnitus and unilateral hearing loss.

Vestibular findings were also comparable between groups. vHIT gains were largely preserved, with only isolated canal abnormalities and no significant between-group differences. HST responses were similar, while VIN was positive in 16.7% of patients with atelectasis and 28.6% of those without atelectasis (*p* = 0.60). Positional findings were comparable, including the distribution of geotropic and apogeotropic nystagmus in the lateral decubitus position. Kinetic VOR gains were similar at both 0.1 Hz (0.39 vs. 0.40; *p* = 0.66) and 0.64 Hz (0.42 vs. 0.39; *p* = 0.90). Step-test time constants were within normal limits in all patients. Cumulative caloric reflectivity was 49.2 in patients with atelectasis and 59.2 in those without atelectasis (*p* = 0.60), with values remaining within the normal range in both groups. None of these between-group differences reached statistical significance.

## Discussion

Persistent apogeotropic positional nystagmus of the lateral SCC represents a complex vestibular disorder that poses considerable diagnostic and therapeutic challenges. It is characterized by persistent positional disturbances, despite appropriately performed therapeutic maneuvers and in association with atypical nystagmus features. The forms (apogeotropic or geotropic) and durations (exceeding 1 min or even non-fatiguing) are not fully explained by the classical pathophysiology of canalithiasis. Therefore, the international definition of BPPV is not met (2).

The rationale of this study was that persistent apogeotropic positional nystagmus of the lateral SCC, when unresponsive to repositioning maneuvers and not explained by central neurological disease, may involve mechanisms distinct from typical canalithiasis. We initially considered several non-exclusive hypotheses, including cupular or canal-related mechanisms, endolymphatic pressure disorders such as hydrops, and vestibular atelectasis. Clinical, electrophysiological, and radiological assessments revealed only rare endolymphatic hydrops and no evidence of central neurological disease, whereas vestibular atelectasis was identified in a substantial proportion of patients with interpretable delayed 3D-FLAIR MRI. However, patients with and without atelectasis did not differ significantly in their clinical, audiological, or vestibular findings. These results document the coexistence of vestibular atelectasis and persistent apogeotropic positional nystagmus but do not establish a causal relationship. Overall, this clinical phenotype appears heterogeneous and may involve several mechanisms.

These findings provide new epidemiological, electrophysiological, and radiological data on a poorly characterized subgroup of persistent apogeotropic positional vestibular disorders.

Existing studies on alBPPV remain limited, mainly comprising small case series with heterogeneous definitions of atypical positional vertigo (5, 6). In our study, alBPPV was defined pragmatically using clinical and VNG criteria, reflecting routine clinical practice: typical canalithiasis is usually recognized and treated effectively with repositioning maneuvers, whereas persistent atypical positional nystagmus without evidence of central neurological disease remains diagnostically and therapeutically challenging. This approach was intended to focus on the group of patients who most closely reflect the unresolved difficulties encountered in daily vestibular practice.

The first notable finding was the duration of diagnostic and therapeutic delays in our cohort (6.8 years). This delay may reflect the diagnostic difficulty of persistent atypical positional nystagmus, the complexity of interpreting vertigo and nystagmus patterns, and the under-recognition of sustained non-response to repositioning maneuvers, which was observed in all patients in our cohort.

The cohort was predominantly female and consisted mainly of older patients, with cardiovascular comorbidities reported in nearly half of cases. The frequency of cardiovascular history was consistent with the age of the cohort and was considered a descriptive finding rather than a specific explanatory factor. One-third of the patients had DHI scores >40 and most lacked auditory deficits. Electrophysiological testing identified pseudospontaneous nystagmus in 22% of cases, similar to the prevalence of approximately 20% previously reported (7). The high-frequency vestibuloocular reflexes (vHIT) were preserved.

Several etiological hypotheses have been proposed since the first descriptions of horizontal canal BPPV and its positional nystagmus variants by Pagnini et al. (3). The persistent apogeotropic positional nystagmus observed in our cohort may be related to a heavy cupula mechanism or continuous stimulation by otoconial debris within the lateral SCC or lateral canal obstruction causing negative endolymphatic pressure between the obstructed region and the cupula (8). Canalithiasis involving the ampullary arm of the lateral SCC has also been proposed as a potential mechanism for apogeotropic positional nystagmus in horizontal canal BPPV, although this hypothesis remains debated and has not been directly demonstrated physiologically or radiologically.

However, several findings in our cohort argue against a purely canalithiasis-based or fixed canal obstruction mechanism. The largely preserved vHIT gains, with only isolated canal abnormalities and the absence of a consistent caloric hypofunction pattern do not support a fixed obstructive mechanism. Low-frequency testing revealed caloric hypofunction in 47% of cases, consistent with the findings of Kim et al. (9), but other vestibular investigations did not reveal a consistent peripheral or central vestibular deficit pattern. Electrocochleography and DPOAE phase-shift analysis showed no significant pressure abnormalities. Brain and contrast-enhanced cerebellopontine angle MRI showed no central nervous system or retrocochlear lesions.

Moreover, the persistent, frequently bilateral, and poorly fatigable nystagmus observed in our cohort appears difficult to reconcile with a purely canalithiasis-based mechanism. These features instead suggest more complex or heterogeneous vestibular mechanisms. While some patients meet cupulolithiasis criteria, others present with bilateral apogeotropic nystagmus, inconsistent with Ewald’s second law. In certain cases, nystagmus evolves over time, shifting from apogeotropic to geotropic or disappearing entirely despite persistent symptoms. However, only a minority of our patients developed a geotropic form during follow-up vestibular assessment, whereas most continued to exhibit persistent apogeotropic positional nystagmus. This temporal variability appears inconsistent with the expected course of typical horizontal canal BPPV and may support the hypothesis of a distinct peripheral vestibular disorder in at least a subset of patients.

In some patients, these fluctuations may be explained by pressure-related conditions, such as Menière’s disease. Endolymphatic pressure fluctuations can cause BPPV through the release of otoconia and/or sensitization of the cupulae (10). However, no patients exhibited low-frequency hearing loss, thereby arguing against this diagnosis (11). Furthermore, electrocochleography, distortion product analysis, and VNG did not reveal pressure abnormalities. The rate of hydrops observed on delayed 3D-FLAIR inner ear MRI was low despite a mean symptom duration of 6.8 years.

Although no clinical or radiological findings supported a central cause in our cohort, cerebrovascular disease and vestibular migraine remain important differential diagnoses in patients with persistent atypical positional nystagmus. Cerebellar infarction or lesions involving the dorsolateral fourth ventricular region or dorsal vermis may produce apogeotropic positional nystagmus, sometimes with few additional neurological signs, and early MRI may occasionally be falsely reassuring (12–14). Vestibular migraine may also present with atypical positional or head-shaking nystagmus during the acute or peri-attack phase (15–17). In our cohort, however, no patient had clinical or radiological evidence of a central vascular lesion or met the Bárány Society criteria for vestibular migraine (18). Comprehensive neurological assessment and brain MRI, with repeated evaluation when clinically indicated, therefore remain essential before considering a peripheral vestibular mechanism. Our cohort does not meet the criteria for central positional paroxysmal vertigo (CPPV) (19).

Among patients with interpretable delayed 3D-FLAIR inner ear MRI, vestibular atelectasis was identified in 61.9% and was predominantly bilateral. The saccule was the most frequently affected, although utricular involvement was also observed.

Nevertheless, the origin of atelectasis remains unclear. Initially described by Schuknecht (20) in 1988, its rediscovery using 3D-FLAIR MRI was more recent, particularly in cases with bilateral vestibular involvement (21). Few studies have evaluated vestibular atelectasis using delayed 3D-FLAIR inner ear MRI, and its occurrence in patients with persistent apogeotropic positional nystagmus involving the lateral SCC remains poorly documented. Vestibular atelectasis was identified in a substantial proportion of patients with interpretable imaging. However, its relationship with positional nystagmus remains uncertain, and the present findings do not establish a causal relationship.

Vestibular atelectasis was not identified in all patients, suggesting that it may represent only one of several mechanisms underlying persistent apogeotropic positional nystagmus. One possible hypothesis is that vestibular atelectasis may reflect a progressive or fluctuating membranous labyrinth process, with early biomechanical alterations preceding radiologically visible collapse. In some patients, subtle or early abnormalities may therefore remain below the detection threshold of delayed 3D-FLAIR MRI performed at 1.5 Tesla. These considerations further support the possibility that alBPPV represents a pathophysiologically heterogeneous condition.

Vestibular atelectasis may alter the morphology of the macular receptors and labyrinthine pressure. The resulting deformation of the membranous labyrinth could generate traction forces on the cupulae and modify their mechanical properties or effective density. These changes could hypothetically contribute to the chronic and irreducible nature of the positional nystagmus. This mechanism may also be consistent with a “floating labyrinth” model, in which vestibular collapse alters labyrinthine mechanics and fluid dynamics, potentially increasing gravity-dependent sensitivity and contributing to persistent direction-changing positional nystagmus (22). The predominantly bilateral distribution of vestibular atelectasis could hypothetically contribute to altered and asymmetric gravity-dependent cupular responses on either side. Such bilateral labyrinthine mechanical changes might also contribute to the persistence of positional nystagmus and the lack of response to standard repositioning maneuvers. This hypothesis is consistent with the observed clinical pattern, although it requires confirmation in dedicated prospective studies. Moreover, the low prevalence of endolymphatic hydrops, the radiological changes involving the vestibular end organs, and the prolonged clinical course observed in this cohort may be consistent with a chronic membranous labyrinth disorder, in which vestibular atelectasis could represent one possible morphological expression (23).

No significant epidemiological, clinical, audiological, or vestibular differences were identified between patients with and without atelectasis. Nevertheless, the frequent detection of vestibular atelectasis in patients with interpretable delayed 3D-FLAIR MRI remains a noteworthy radiological finding. It may identify a previously underrecognized inner ear abnormality in this clinical population and could contribute to the pathophysiology in a subset of patients. One previous study described the clinical and vestibular features of patients with unilateral vestibular atelectasis identified on inner ear MRI (24). In that cohort, positional vertigo was present in 41% of cases, pseudospontaneous nystagmus in 25%, and positional nystagmus could be either geotropic or apogeotropic. Vertigo typically had a sudden onset and lasted less than 20 min, while otological symptoms and auditory asymmetry were reported in 50 and 40% of cases, respectively. Vestibular testing revealed reduced VOR gain in at least one SCC on vHIT and ipsilateral caloric hypofunction in 60% of cases. However, that population was not selected on the basis of persistent apogeotropic positional nystagmus, and its overall clinical and vestibular profile differed from that observed in our cohort.

### Future research

Future prospective multicenter studies should include larger cohorts of patients with atypical, non-resolving, or frequently recurrent BPPV-like positional vertigo. Combining standardized vestibular testing, longitudinal follow-up, and high-resolution delayed 3D-FLAIR inner ear MRI could help identify distinct radiological patterns, assess symptom and nystagmus fluctuations, evaluate the effect of repeated maneuvers or vestibular rehabilitation, and distinguish otoconial disorders from broader membranous labyrinth or central mechanisms.

### Strengths and limitations

The main strengths of this study include the homogeneous clinical phenotype of the included patients, the standardized vestibular assessment performed by the same team, and the multidisciplinary review of VNG recordings and delayed 3D-FLAIR inner ear MRI.

This retrospective, single-center study has inherent limitations, including the absence of an *a priori* power analysis and a small but clinically and electrophysiologically homogeneous sample size. Therefore, the results should be interpreted as exploratory. Potential confounding factors include the retrospective design, variable delay between symptom onset, vestibular testing, and MRI, previous repositioning maneuvers or vestibular rehabilitation, comorbidities, and the fluctuating nature of positional nystagmus. Because the DHI was assessed only once, it could not be used to evaluate changes over time or the effect of repositioning maneuvers or vestibular rehabilitation.

Delayed 3D-FLAIR inner ear MRI was evaluable in 21 of the 32 included patients. In this retrospective real-world cohort, delayed inner ear MRI was proposed as part of the diagnostic work-up for atypical, persistent, or treatment-resistant vestibular presentations, after routine brain MRI had excluded a central cause. Nine patients declined or could not complete this additional examination because of claustrophobia, and two examinations were uninterpretable because of motion artefacts. These missing data were therefore primarily related to examination acceptability and technical feasibility rather than to the expected radiological findings. Nevertheless, the possibility of selection bias cannot be entirely excluded, and the observed frequency of vestibular atelectasis should be interpreted as applying only to patients with evaluable imaging.

The absence of a comparison group of patients with typical, maneuver-responsive BPPV undergoing the same delayed 3D-FLAIR inner ear MRI protocol limits our ability to determine whether vestibular atelectasis is specifically associated with persistent apogeotropic positional nystagmus. The frequency of vestibular atelectasis in patients with typical BPPV remains uncertain because comparative studies using the same delayed 3D-FLAIR inner ear MRI protocol are lacking. Comparative studies using standardized imaging protocols in both persistent apogeotropic positional nystagmus and typical BPPV would therefore be particularly informative.

Bias was minimized through predefined inclusion criteria, standardized assessment procedures, and multidisciplinary review of VNG and MRI findings. Nevertheless, the interpretation of nystagmus may involve a degree of subjectivity and the assessment of delayed 3D-FLAIR inner ear MRI remains qualitative, as no validated quantitative criteria are currently available. MRI examinations were performed using a 1.5- rather than a 3-Tesla system. Although the spatial resolution is lower at 1.5 T, the identification of endolymphatic hydrops is not precluded, as previously reported (25). Vestibular atelectasis can be detected at this field strength.

The absence of o-VEMP due to technical limitations during the study period, together with the difficulty in interpreting c-VEMP data, are key study limitations. The c-VEMP responses were frequently absent and predominantly bilateral, preventing reliable side-specific interpretations. These data were excluded to avoid misleading conclusions. The integrity of superior and inferior vestibular nerves could therefore not be fully assessed, although the saccule is the primary macula affected by atelectasis. Previous studies have reported that 54–61% of patients with saccular collapse exhibit electrophysiological deficits (21, 26).

## Conclusion

Persistent irreducible apogeotropic positional nystagmus of the lateral SCC that does not respond to repositioning maneuvers appears to represent a heterogeneous vestibular disorder. Among patients with interpretable delayed 3D-FLAIR MRI, vestibular atelectasis was commonly observed but was not associated with a specific clinical, audiological, or vestibular profile. These findings document the coexistence of vestibular atelectasis and persistent apogeotropic positional nystagmus but do not establish a causal or disease-specific relationship. Prospective, controlled, and longitudinal studies are required to determine its clinical and pathophysiological significance in this population.

## Data Availability

The raw data supporting the conclusions of this article will be made available by the authors, without undue reservation.
